# Elevation of Eosinophil-Derived Neurotoxin in Plasma of the Subjects with Aspirin-Exacerbated Respiratory Disease: A Possible Peripheral Blood Protein Biomarker

**DOI:** 10.1371/journal.pone.0066644

**Published:** 2013-06-21

**Authors:** Seung-Woo Shin, Jong Sook Park, Choon-Sik Park

**Affiliations:** Genome Research Center for Allergy and Respiratory Diseases, Division of Allergy and Respiratory Medicine, Soonchunhyang University Bucheon Hospital, Bucheon, Gyunggi-do, Republic of Korea; NIAID, United States of America

## Abstract

Aspirin-exacerbated respiratory disease (AERD) remains widely underdiagnosed in asthmatics, primarily due to insufficient awareness of the relationship between aspirin ingestion and asthma exacerbation. The identification of aspirin hypersensitivity is therefore essential to avoid serious aspirin complications. The goal of the study was to develop plasma biomarkers to predict AERD. We identified differentially expressed genes in peripheral blood mononuclear cells (PBMC) between subjects with AERD and those with aspirin-tolerant asthma (ATA). The genes were matched with the secreted protein database (http://spd.cbi.pku.edu.cn/) to select candidate proteins in the plasma. Plasma levels of the candidate proteins were then measured in AERD (n = 40) and ATA (n = 40) subjects using an enzyme-linked immunosorbent assay (ELISA). Target genes were validated as AERD biomarkers using an ROC curve analysis. From 175 differentially expressed genes (*p*-value <0.0001) that were queried to the secreted protein database, 11 secreted proteins were retrieved. The gene expression patterns were predicted as elevated for 7 genes and decreased for 4 genes in AERD as compared with ATA subjects. Among these genes, significantly higher levels of plasma eosinophil-derived neurotoxin (RNASE2) were observed in AERD as compared with ATA subjects (70(14.62∼311.92) µg/ml vs. 12(2.55∼272.84) µg/ml, *p*-value <0.0003). Based on the ROC curve analysis, the AUC was 0.74 (*p*-value = 0.0001, asymptotic 95% confidence interval [lower bound: 0.62, upper bound: 0.83]) with 95% sensitivity, 60% specificity, and a cut-off value of 27.15 µg/ml. Eosinophil-derived neurotoxin represents a novel biomarker to distinguish AERD from ATA.

## Introduction

Aspirin-exacerbated respiratory disease (AERD) refers to the development of bronchoconstriction and nasal manifestations in asthmatic individuals following the ingestion of aspirin and/or other nonsteroidal anti-inflammatory drugs (NSAIDs) [1], [2]. Aspirin represents the most commonly used medication for pain control, pain prophylactics, and the primary and secondary prevention of coronary artery disease or other vascular diseases [3]. Recently, aspirin hypersensitivity has attracted a great deal of attention due to its association with increased asthma severity, including life threatening asthma attacks and the possible remodeling of both the upper and lower airways [4]. In patients requiring emergency mechanical ventilation, the prevalence of aspirin intolerance was reported to be 24.3% [5]. Picado et al. reported the occurrence of life-threatening asthma attacks requiring mechanical ventilation in 14% of adults with aspirin intolerance [6]. The high incidence of severe asthma attacks may originate from underdiagnosis in asthmatics, due to insufficient awareness of the relationship between aspirin ingestion and asthma exacerbation. It is of note that a reported 15–30% of patients are entirely unaware that they suffer from aspirin intolerance, with only provocation tests ultimately revealing their hypersensitivity [7], [8]. Thus, the identification of aspirin hypersensitivity is essential to avoid serious aspirin complications.

A definitive diagnosis can only be established through provocation tests using incremental doses of aspirin [9]. Although nasal or bronchial provocation with lysine-ASA may represent a valuable alternative diagnostic tool, oral aspirin challenge (OAC) is the gold standard to confirm the diagnosis. However, OAC is a time-consuming procedure and serious complications occur in some cases [10]. Thus, the development of non-invasive methods is necessary to allow for a simple diagnosis that circumvents the unexpected complications of aspirin use in susceptible patients.

The identification of disease-specific genes allows for early disease detection, prognosis, and treatment [11]. High-throughput microarrays have become an important tool in functional genomics studies and are commonly used to address various biological questions. Large-scale, high-throughput, and whole-genome studies have been performed to understand the genomic contribution of asthma, and to develop specific biomarkers for diagnosis. Recently, we performed a whole-genome microarray for the transcriptome in peripheral blood mononuclear cells (PBMCs) obtained from subjects with AERD and ATA. Differential gene expression profiles in the PBMCs from these subjects were identified [12]. The data in our previous publication [12] have been deposited in NCBI’s Gene Expression Omnibus [13] and are accessible through GEO Series accession number GSE45847 (http://www.ncbi.nlm.nih.gov/geo/query/acc.cgi?acc=GSE45847). Based on this analysis, 176 genes showed significantly altered mRNA expression profiles in AERD as compared with ATA. This prompted us to determine which of these genes products are secreted into the peripheral blood and may therefore act as a biomarker, due the ease of obtaining plasma protein samples.

In this study, we integrated genes displaying altered mRNA expression profiles in PBMCs based on the gene-chip data (*p*-value <0.0001) to the secreted protein database (http://spd.cbi.pku.edu.cn/) in order to identify candidate plasma biomarkers that may be useful as diagnostic markers of AERD. We then quantified plasma protein levels by enzyme-linked immunosorbent assay (ELISA) and assessed their discriminative ability using ROCs between AERD and ATA groups.

## Methods

### Ethics Statement

This study was approved by the ethics committee on BioBank of the University of SHOONCHUNHANG, Korea (NO: schbc-biobank-2011-015) and complied with the ethical standards laid down in the 1964 Declaration of Helsinki. All participants read and signed an informed consent statement. Following the ethical standards of the local committee, each participant received detailed information regarding the purpose of the study at the end of the experimental session. All participants’ data were analyzed and reported anonymously.

### Subjects

Subjects were recruited from the Genome Research Center for Allergy and Respiratory Diseases in the Soonchunhyang University Hospital. All subjects were Korean. All patients met the definition of asthma as defined in the Global Initiative for Asthma (GINA) guidelines [14]. Each patient showed airway reversibility, as documented by a positive bronchodilator response greater than 15%, an increase in the forced expiratory volume in one second (FEV1), and/or airway hyperreactivity to >10 mg/ml methacholine. The subjects were skin-prick tested for 24 common inhalant allergens, including house dust mites, *Alternaria, Aspergillus*, pollens, dogs, cats, and cockroaches. Atopy was defined as a wheal reaction to the allergen extract that was equal to or greater than 3 mm in diameter to that of histamine (1 mg/ml). Total IgE was measured using the UniCAP system (Pharmacia Diagnostics, Uppsala, Sweden). No exacerbation or systemic steroid treatment within 6 weeks of the study was performed.

An oral aspirin challenge (OAC) was performed with increasing doses of aspirin using methods slightly modified from those previously described [9], [15]. Briefly, patients with a history of aspirin hypersensitivity were administered 30 mg orally. Respiratory and nasal symptoms, blood pressure, external signs (urticaria and angioedema), and FEV_1_ were documented every 30 min for a period of 1.5 h. In the absence of any symptoms or signs that suggested an adverse reaction after 1.5 h, increasing doses of aspirin (60, 100, 300, and 400 mg) were administered and the same measurements were repeated every 1 h until the patient developed a reaction. Patients with no history were initiated onto 100 mg of aspirin and the dose gradually increased to 200, 350, and 450 mg until a reaction developed. If no reaction occurred 4 h after the final dose, the test was deemed negative. Aspirin-induced bronchospasm, reflected by a decline (%) in FEV_1_, was calculated as the pre-challenge FEV_1_ minus the post-challenge FEV_1_ divided by the pre-challenge FEV_1_. OAC reactions were categorized into two groups: (1) 15% or greater decrease in FEV_1_ or nasal reactions (aspirin-exacerbated respiratory diseases [AERD]) or (2) less than 15% decrease in FEV_1_ without naso-ocular or cutaneous reactions (aspirin-tolerant asthma [ATA]). Plasma was collected from a heparinized blood sample taken prior to aspirin challenge and stored in the hospital biobank. The clinical profiles for ATA and AERD patients are summarized in Table 1.

**Table 1:** Clinical profiles of the study subjects.

	ATA	AERD
Number of subjects (n)	40	40
Age [year, median (Range)]	50(26∼79)	57(27∼78)
Onset age of asthma [year, median (range)]	38(7∼72)	47(1∼68)
Sex (n, male/female)	20/20	12/28
Current smoker/ex-smoker (%)	22.5/17.5	15.0/10.0
Decline (%) of FEV_1_ by aspirin provocation¥	−7.0(−15∼2)	27.0(17∼82)*
Body mass index (kg/m^2^)	25.8±3.6	23.9±3.0*
Blood eosinophils percent¥	4.3(0.9∼28.9)	5.8(0.7∼23.6)
FEV_1_, % predicted	81.9±20.4	84.1±21.9
PC20 methacholine (mg/ml)¥	1.8(0.2∼11.3)	2.8(0.1∼19.4)
Positive rate of skin test (%)	23(57.5)	20(50.0)

* : *p*-value <0.05 for the difference between AERD and ATA.

¥ Because the data is not normally distributed, Mann-Whitney U is used and median (range) were represented.

### Integration of Candidate Genes for AERD

We selected candidate genes (*p*-value *<*0.0001) between the AERD and ATA groups using our previous gene-chip data analysis [12]. The selected genes were integrated into the secretory protein database: SPD (http://spd.cbi.pku.edu.cn/). The SPD database contains a total of 18256 secreted proteins based on the UniProt Knowledgebase Release 7.0, Reference Sequence Release 15.0, and Ensembl Release 39 [16].

### Measurement of the Eosinophil-derived Neurotoxin Levels

From the identified candidate genes, we assessed the plasma levels of the eosinophil-derived neurotoxin using a quantitative Human Ribonuclease A2 ELISA Kit (Uscn Life Science Inc, Wuhan, CHINA). The lower limit of the eosinophil-derived neurotoxin detection was 2.554 µg/ml. Any values below this limit were assumed as zero for the purposes of statistical analysis. The inter- and intra-assay coefficients of variance were below 10%.

### Statistical Analyses

Statistical analyses were performed using R software (ver. 2.13.1; http://www.r-project.org/). For sex, smoker and positive rate of skin test variable that were summarized as frequencies, the fisher’s exact test were applied. For age, onset age of asthma, BMI, FEV1 variable, they satisfied the normality of distribution and the equal variance assumption. So the *t*-test was performed. Because peripheral blood eosinophils (%), PC20 and decline of FEV1 by aspirin provocation didn’t satisfy the normality assumption, Mann-Whitney U test was performed. The Mann-Whitney U was also applied to compare plasma protein levels between AERD and ATA subjects. A multiple logistic regression (MLR) analysis was performed to calculate ROC curves and AUCs. [17], [18]. Correlations with other clinical values were also assessed. By the result of normality test, either Pearson (age, onset age of asthma, BMI, FEV1) or Spearman (blood eosinophils (%), PC20 and FEV1) method was used. The clinical and laboratory values are expressed as arithmetic mean (including standard deviation) or median (including range) by the result of distribution. The statistical significance was defined as a *p*-value <0.05.

## Results

### Patient Characteristics

Eighty asthmatic patients were classified into two groups (AERD vs. ATA) based on the aspirin challenge test. Age, sex, smoking, BMI, blood eosinophil %, FEV1 and PC20 methacholine were matched between the AERD and ATA subjects. The % decrease of FEV_1_ due to aspirin challenge (*p*-value = 1.398e-14) and BMI (*p*-value = 0.01235) differed between the two groups (Table 1).

### Candidate Genes for AERD

Among the 318 differentially expressed genes between the AERD and ATA groups using our gene-chip data analysis [12], we selected a total of 175 genes with a *p*-value less than 0.0001 and queried them with the secreted protein database (SPD). Among the 175 genes, a total of 11 genes were identified as secreted proteins (Table 2). The fold changes ranged from 0.71 to 10.07. The AERD group had the highest fold change of eosinophil-derived neurotoxin gene expression when compared to that of ATA group. This prompted us to measure plasma levels of eosinophil-derived neurotoxin.

**Table 2:** Candidate proteins identified from the secreted protein database.

Gene Symbol	P-value	Fold change	Description
BRD9	0.0000016044	0.73	Bromodomain containing 9
APOM	0.0000031894	0.71	Apolipoprotein M
BMP4	0.0000083773	1.60	Bone morphogenetic protein 4
COL6A3	0.0000097721	4.87	Collagen type VI alpha 3
PIK3CD	0.0000119903	0.85	Phosphoinositide-3-kinase catalytic delta polypeptide
C1QA	0.0000153412	2.40	Complement component 1q subcomponent A chain
ADAMTS13	0.0000360935	2.14	ADAM metallopeptidase with thrombospondin type 1 motif 13
DNASE2	0.0000360935	1.42	Deoxyribonuclease II, lysosomal
RCN1	0.0000488112	0.80	Reticulocalbin 1, EF-hand calcium binding domain
RNASE2	0.0000705104	10.07	Ribonuclease RNaseA family 2 (liver eosinophil-derived neurotoxin)
NTN2L	0.0000802616	1.92	Netrin 2-like (chicken)

### Eosinophil-derived Neurotoxin Plasma Levels in ATA vs. AERD Subjects and Correlation with Aspirin-induced Bronchospasm

**Figure 1:**
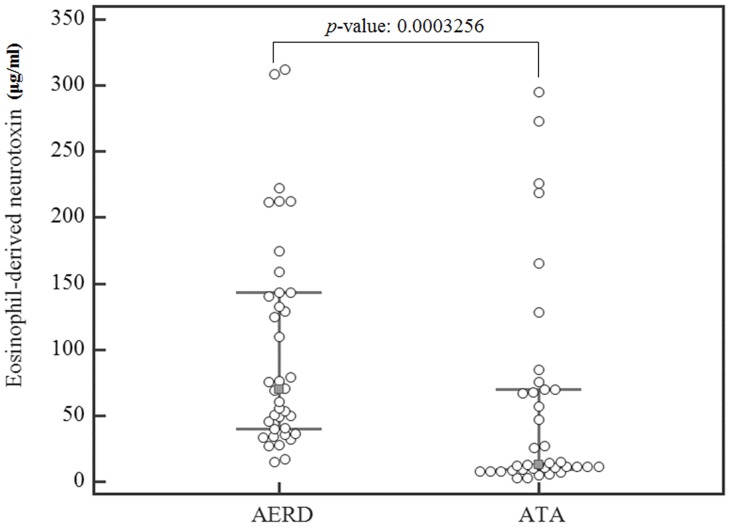
Box plot of the eosinophil-derived neurotoxin expression levels in AERD and ATA groups as assessed by ELISA (Error bar: 25∼75 percentiles).

When plasma eosinophil-derived neurotoxin levels were quantified by ELISA, significantly higher levels were observed in subjects with AERD (n* = *40) as compared with ATA subjects (n* = *40) (70(14.62∼311.92) µg/ml vs. 12(2.55∼272.84) µg/ml, p<0.0003, Fig. 1). Plasma levels of eosinophil-derived neurotoxin were significantly correlated with % fall decrease of FEV_1_ by aspirin challenge in all subjects (*p*-value = 0.024, r = 0.252, Table 2), but did not correlate with age, asthma onset, basal FEV_1_, BMI and PC20 methacholine (Table 3). Peripheral blood eosinophil % tended to correlate with plasma eosinophil-derived neurotoxin levels (r = 0.207, *p*-value = 0.067).

**Table 3:** Correlation of eosinophil-derived neurotoxin levels with other clinical variables.

	% fall*	Age	Onset of asthma	FEV_1_	BMI	Eosinophil(%)	PC20
Correlation coefficient	0.252	−0.052	0.003	−0.074	−0.053	0.207	−0.098
*p*-value	*0.024*	0.647	0.979	0.516	0.64	0.067	0.41
N	80	80	80	80	80	79	73

* % decrease indicates decline (%) of FEV1 due to aspirin treatment.

### Logistic Regression Analysis to Predict AERD using Eosinophil-derived Neurotoxin and the ROC Curve Analysis

**Figure 2:**
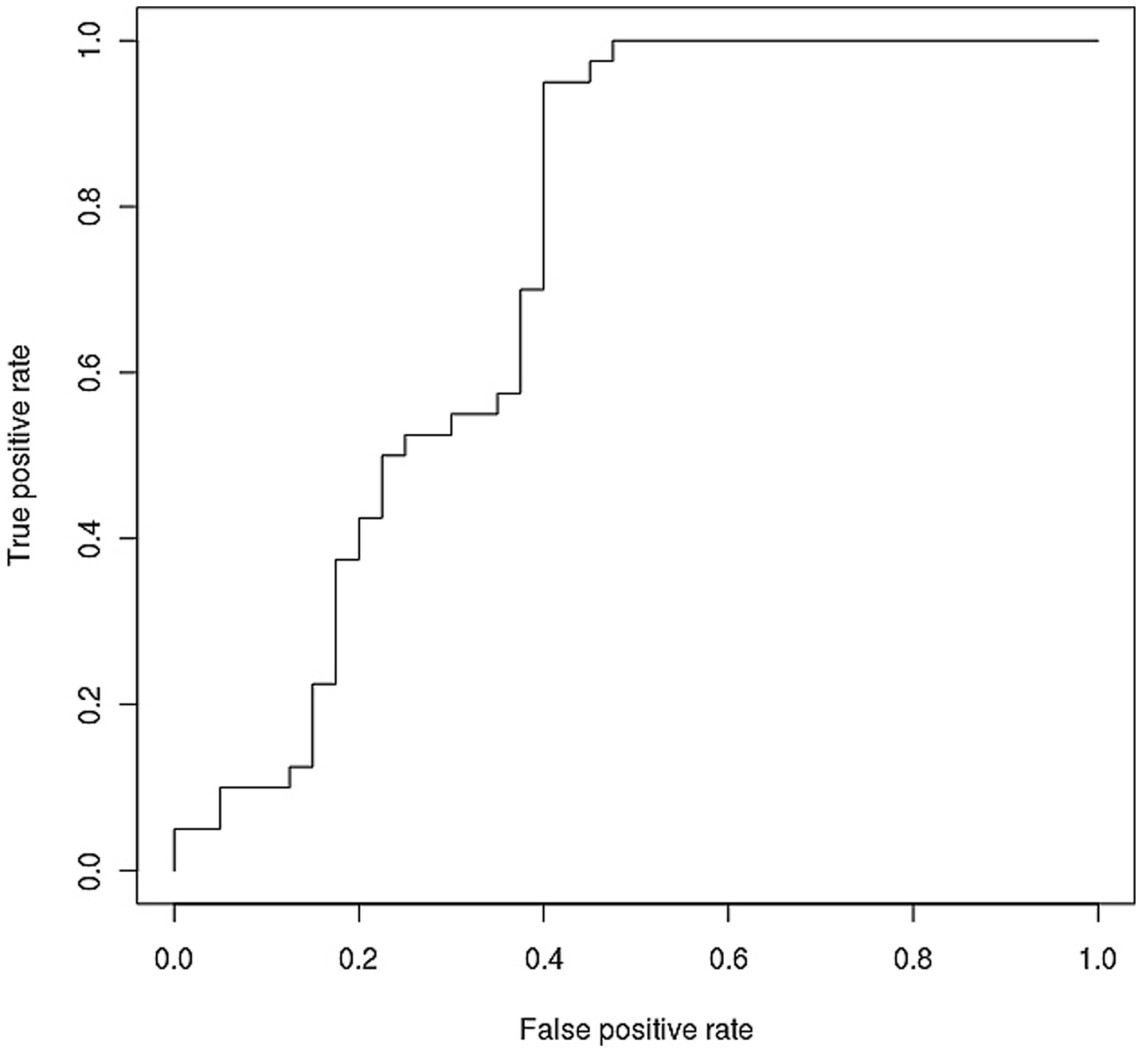
ROC curve of plasma eosinophil-derived neurotoxin.

To determine the power of eosinophil-derived neurotoxin as a predictive biomarker for AERD, we applied a multiple linear regression (MLR) analysis using the measured plasma eosinophil-derived neurotoxin levels. Fig. 2 displays the results of the ROC curve analysis aimed at assessing the ability of eosinophil-derived neurotoxin to assess diagnostic accuracy. The AUC of the plasma eosinophil-derived neurotoxin ROC curve was 0.74 (*p*-value = 0.0001, asymptotic 95% confidence interval [lower bound: 0.62, upper bound: 0.83], cut-off value: 27.15 µg/ml, sensitivity: 95%, specificity: 60%).

## Discussion

From the secreted protein database, we identified 11 genes whose mRNA expression profiles differed between AERD and ATA patients based on a gene-chip data analysis. Among them, we measured the plasma levels of eosinophil-derived neurotoxin, which we identified as a biomarker to predict AERD. Eosinophils contain four principal cationic proteins: major basic protein (MBP), eosinophil-derived neurotoxin (EDN: RNASE2), eosinophil cationic protein (ECP), and eosinophil peroxidase (EPO) [19].

Since eosinophils may be associated with severe asthma, and a marker associated with eosinophils may be related with severity rather than aspirin sensitivity, FEV1 and PC20 methacholine, blood eosinophil %, age, sex, smoking, BMI were matched between the AERD and ATA subjects. Although, the plasma levels of eosinophil-derived neurotoxin did not correlate with the % of eosinophils in the blood, indicating that the increase of plasma eosinophil-derived neurotoxin in subjects with AERD may be due to the enhanced activation of eosinophils compared to ATA subjects, there was a trend towards significance.

Differential levels of eosinophil activation have been observed between the airways and peripheral blood. The proportion of hypodense eosinophils in patients with asthma was significantly greater than that of normal donors. The concentration of plasma eosinophil granule MBPs correlated with the numbers of peripheral blood eosinophils and hypodense eosinophils [20]. Serum ECP concentrations in asthma patients were significantly higher than those of COPD patients and healthy subjects. In addition, a significant inverse correlation between the serum ECP concentration and % FEV1 was reported [21].

Eosinophil-derived neurotoxin possesses multiple functions. It is a powerful neurotoxin in rabbits and guinea pigs and causes paralysis in experimental animals when injected intrathecally [20], [22]. Eosinophil-derived neurotoxin is also a potent ribonuclease [23] and possesses weak helminthotoxic activity [24], [25]. In addition, Eosinophil-derived neurotoxin has been implicated in cell damage and neurotoxicity [26].

In the present study, we demonstrate that the sensitivity and specificity of plasma Eosinophil-derived neurotoxin levels are 95% and 60%, respectively, for the prediction of AERD using an MLR analysis. Although the result doesn’t show high specificity, it shows quit high sensitivity. The AUC of the ROC curve was 0.74, which represents a high diagnostic accuracy. Thus, we propose that eosinophil-derived neurotoxin levels as determined by ELISA can be utilized as a biomarker to distinguish between AERD and ATA subjects.

Several studies aimed at developing non-invasive methods for the simple diagnosis of AERD have been attempted. Mascia, et al reported a validated computed tomography (CT) scan– based scoring system to distinguish AERD from ATA [27]. The extent of hyperplasia on CT scan and the presence of nasal polyps are very useful markers of AERD. Patients with AERD had significantly more severe sinusitis based on sinus CT score, and probability of AERD may be predicted based on sinus CT score (receiver operating characteristic area under the curve = 0.85). In peripheral blood, flow cytometric determination of basophil activation has been proposed for the *in vitro* diagnosis of NSAID hypersensitivity syndrome [28]. Galectin-10 mRNA [29] and plasma eotaxin 2 [30] have also been found to be elevated in subjects with AERD as compared with ATA. Several proteomic candidates have been characterized as associated with an increased risk of AERD [31]. Urine leukotriene E4 and plasma 9 alpha 11 beta PGF(2) in exhaled breath condensate displayed high sensitivity and specificity for discriminating between the two groups [32]. Many genetic variants in the arachidonate pathways [33], [34] and immune and inflammatory pathways [15], [35], [36] also appear to be involved in the development of AERD. We evaluated the diagnostic value of clinical parameters including the well-known manifestations of aspirin hypersensitivity history, nasal polyposis, and chronic sinusitis in the prediction of aspirin hypersensitivity in asthma. In the AERD group, there were more females than males in our previous report [12] and other ones [2], [7]. In the present study, female was dominant in AERD group. Among them, a history of aspirin hypersensitivity displayed the best positive and negative predictive values for a positive aspirin challenge test with an overall accuracy of 88.2% [8]. However, false positive and negative rates remained high. Thus, further studies are required to identify biomarkers that display superior results to these clinical parameters.

### Conclusion

In conclusion, using the secreted proteins of 11 genes that display differential mRNA expression levels between ARED and ATA, we identified eosinophil-derived neurotoxin as a biomarker that differentiates between AERD and ATA with a high discriminative power. The AUC of the ROC curve for eosinophil-derived neurotoxin was 0.74 (*p*-value: 0.0003, asymptotic 95% confidence interval [lower bound: 0.62, upper bound: 0.85]). The sensitivity and specificity were 90% and 60%, respectively. Thus, eosinophil-derived neurotoxin represents a gene marker from PBMCs that may be diagnostically useful for the prediction of AERD.
